# Benzoyl­methyl pyridine-4-carboxyl­ate

**DOI:** 10.1107/S1600536808014086

**Published:** 2008-05-17

**Authors:** Jian-Nan Guo, Bo-Chao Zhang, Yi Jin, Guo Tang, Yu-Fen Zhao

**Affiliations:** a The Key Laboratory for Chemical Biology of Fujian Province, Department of Chemistry, Xiamen University, Xiamen 361005, People’s Republic of China; b Department of Chemistry, Xiamen University, Xiamen 361005, People’s Republic of China

## Abstract

In the crystal structure of the title compound, C_14_H_11_NO_3_, isolated from the reaction of 2-bromo-1-phenyl­ethanone and pyridine-4-carboxylic acid using triethyl­amine as a base to deprotonate the organic acid, the mol­ecular packing is stabilized by C—H⋯π inter­actions involving the phenyl and pyridine rings. The C—C—O—C torsion angle for the linkage between the two carbonyl groups is −80.8 (2)°, and the planes of the phenyl and pyridyl rings form a dihedral angle of 65.8 (1)°.

## Related literature

For related literature, see: Allen *et al.* (1987 ▶); Hendrickson & Kandall (1970 ▶); Pavel *et al.* (1993 ▶).
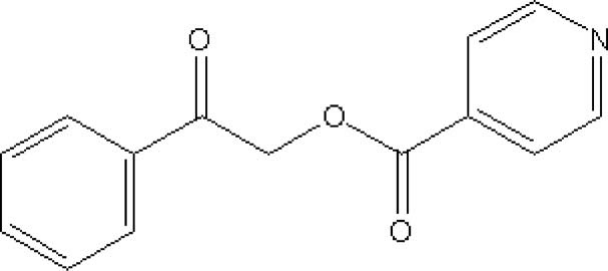


## Experimental

### 

#### Crystal data

C_14_H_11_NO_3_
*M* *_r_* = 241.24
Triclinic, 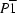

*a* = 8.0863 (6) Å
*b* = 9.2130 (7) Å
*c* = 9.3291 (8) Å
α = 106.738 (7)°
β = 114.495 (8)°
γ = 96.549 (6)°
*V* = 583.52 (8) Å^3^
*Z* = 2
Mo *K*α radiation
μ = 0.10 mm^−1^
*T* = 173 (2) K
0.30 × 0.17 × 0.12 mm

#### Data collection

Bruker APEX CCD diffractometer
Absorption correction: multi-scan (*SADABS*; Bruker, 2001 ▶) *T*_min_ = 0.971, *T*_max_ = 0.988
4945 measured reflections
2011 independent reflections
1099 reflections with *I* > 2σ(*I*)
*R*_int_ = 0.042

#### Refinement

*R*[*F* ^2^ > 2σ(*F* ^2^)] = 0.039
*wR*(*F* ^2^) = 0.074
*S* = 0.82
2011 reflections
163 parameters
H-atom parameters constrained
Δρ_max_ = 0.11 e Å^−3^
Δρ_min_ = −0.13 e Å^−3^

### 

Data collection: *SMART* (Bruker, 2001 ▶); cell refinement: *SAINT* (Bruker, 2001 ▶); data reduction: *SAINT*; program(s) used to solve structure: *SHELXS97* (Sheldrick, 2008 ▶); program(s) used to refine structure: *SHELXL97* (Sheldrick, 2008 ▶); molecular graphics: *ORTEP-3* (Farrugia, 1997 ▶); software used to prepare material for publication: *SHELXL97*.

## Supplementary Material

Crystal structure: contains datablocks I, global. DOI: 10.1107/S1600536808014086/cf2193sup1.cif

Structure factors: contains datablocks I. DOI: 10.1107/S1600536808014086/cf2193Isup2.hkl

Additional supplementary materials:  crystallographic information; 3D view; checkCIF report

## Figures and Tables

**Table 1:** Hydrogen-bond geometry (Å, °)

*D*—H⋯*A*	*D*—H	H⋯*A*	*D*⋯*A*	*D*—H⋯*A*
C4—H4⋯CgB^i^	0.95	3.86	4.766 (3)	160
C2—H2⋯CgB^ii^	0.95	2.89	3.640 (3)	137
C6—H6⋯CgB^iii^	0.95	3.00	3.752 (3)	137
C12—H12⋯CgA^iv^	0.95	2.88	3.572 (3)	130

Symmetry codes: (i) 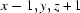; (ii) 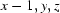; (iii) 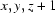; (iv) 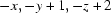. *CgA* and *CgB* are the centroids of the phenyl and pyridine rings respectively.

**Fig. 1.**
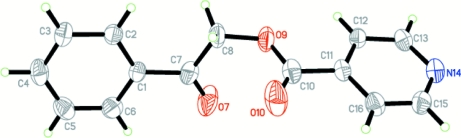
The molecular structure of (I) with the atom-labelling scheme, showing 50% probability displacement ellipsoids. H atoms are drawn as spheres of arbitrary radius.

**Fig. 2.**
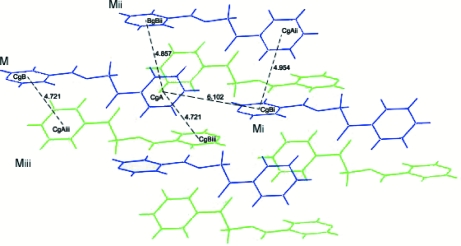
A view of the C—H···π interactions motif of (I). CgA and CgB are the centroids of the benzene and pyridine rings respectively. The C—H···π interactions are shown as dashed lines. The molecules labelled with same color are in one plane. [Symmetry code: (i) x-1, y, z+1; (ii) x-1, y, z; (iii) -x, 1-y, 2-z.]"

## supplementary crystallographic information

### Comment

The title compound was synthesized for a study of protection of the carboxyl group. The phenacyl group has been proved to be an important reagent for protecting carboxyl functions during synthesis in the presence of other esters (Hendrickson *et al.*, 1970).

The title compound (I) was obtained by the reaction of 2-bromo-1-phenylethanone and pyridine-4-carboxylic acid using triethylamine as a base to deprotonate the organic acid. An X-ray crystal structure determination of compound (I) was carried out to determine its conformation. Bond lengths and angles are in agreement with values reported in the literature (Allen *et al.*, 1987).  The torsion angle C7-C8-O9-C10 [-80.8 (2)Å] describes the conformation of the phenyl group with respect to the pyridyl group; the planes of the benzene ring and the pyridine ring form a dihedral angle of 65.8 (1)Å.

In the crystal structure of (I), the phenyl and pyridyl rings make a dihedral angle of 65.8 (1)° and the C7—C8—O9—C10 torsion angle is -80.8 (2)° (Fig. 1). The packing of the aromatic rings is shown in Fig. 2. Two head-to-tail molecules (*M* and *M*^i^) are linked by C—H···π interactions with typical geometry (Pavel *et al.*, 1993), leading to the formation of a linear chain. The distance between CgA and CgB^i^ is 6.102 (3) Å and the angles between the lines through the centroids of the two rings and the normal through CgA is 79.5 (1)° and through CgB^i^ is 76.8 (1)°. The linear chains are further stabilized by other C—H···π interactions (*M* and *M*^ii^; *M*^i^ and *M*^ii^), generating sheets parallel to (010). The corresponding values for the phenyl ring in *M* and the pyridyl ring in *M*^ii^ are 4.857 (3) Å, 69.3 (1)° and 13.7 (1)°. For the phenyl ring in *M*^ii^ and the pyridyl ring in *M*^i^ they are 4.954 (3) Å, 69.4 (1)° and 16.6 (1)°. C—H···π interactions between two sheets (*M* and *M*^iii^) also provide stability for the crystal structure. The corresponding values for the two adjacent aromatic rings in M and M^iii^ are 4.721 (3)Å, 14.5 (1)° and 66.4 (1)°. CgA and CgB stand for the centroids of phenyl and pyridyl rings respectively. [Symmetry codes: (i) *x* - 1, *y*, *z* + 1; (ii) *x* - 1, *y*, *z*; (iii) -*x*, 1 - *y*, 2 - *z*.]

### Experimental

The title compound was prepared by a method based on one described by Hendrickson & Kandall (1970). Triethylamine (1.0 ml, 7.5 mmol) was added dropwise to a mixture of 2-bromo-1-phenylethanone (995 mg, 5 mmol) and pyridine-4-carboxylic acid (615 mg, 5 mmol) in freshly distilled tetrahydrofuran (20 ml) at room temperature under argon and stirred overnight. The precipitate was collected at the pump and washed with ethyl acetate. The filtrate and washings were combined and back-washed successively with 1/3 of the volume each of 10% citric acid, 10% sodium bicarbonate, and water and then dried. Solvent was distilled off *in vacuo* and the residue recrystallized repeatedly from ethyl acetate-petroleum ether, giving 1.04 g (86%) as colourless needles.

### Refinement

The hydrogen atoms were positioned geometrically (C—H = 0.93, 0.98, 0.97 or 0.96Å for aromatic, tertiary, methylene or methyl H atoms respectively) and were included in the refinement in the riding model approximation. The displacement parameters of methyl H atoms were set to 1.5*U*_eq_(C), while those of other H atoms were set to 1.2*U*_eq_(C).

### Crystal data

**Table:**

C_14_H_11_NO_3_	*Z* = 2
*M**_r_* = 241.24	*F*_000_ = 252
Triclinic, *P*1	*D*_x_ = 1.373 Mg m^−^^3^
Hall symbol: -P 1	Mo *K*α radiation λ = 0.71073 Å
*a* = 8.0863 (6) Å	Cell parameters from 1153 reflections
*b* = 9.2130 (7) Å	θ = 2.4–32.7º
*c* = 9.3291 (8) Å	µ = 0.10 mm^−^^1^
α = 106.738 (7)º	*T* = 173 (2) K
β = 114.495 (8)º	Needle, colorless
γ = 96.549 (6)º	0.30 × 0.17 × 0.12 mm
*V* = 583.52 (8) Å^3^	

### Data collection

**Table:**

Bruker APEX CCD diffractometer	2011 independent reflections
Radiation source: fine-focus sealed tube	1099 reflections with *I* > 2σ(*I*)
Monochromator: graphite	*R*_int_ = 0.042
Detector resolution: 16.1903 pixels mm^-1^	θ_max_ = 25.0º
*T* = 173(2) K	θ_min_ = 2.4º
φ and ω scans	*h* = −9→9
Absorption correction: multi-scan(SADABS; Bruker, 2001)	*k* = −10→9
*T*_min_ = 0.971, *T*_max_ = 0.988	*l* = −10→11
4945 measured reflections	

### Refinement

**Table:**

Refinement on *F*^2^	Secondary atom site location: difference Fourier map
Least-squares matrix: full	Hydrogen site location: inferred from neighbouring sites
*R*[*F*^2^ > 2σ(*F*^2^)] = 0.039	H-atom parameters constrained
*wR*(*F*^2^) = 0.074	*w* = 1/[σ^2^(*F*_o_^2^) + (0.0282*P*)^2^] where *P* = (*F*_o_^2^ + 2*F*_c_^2^)/3
*S* = 0.82	(Δ/σ)_max_ < 0.001
2011 reflections	Δρ_max_ = 0.11 e Å^−^^3^
163 parameters	Δρ_min_ = −0.13 e Å^−^^3^
Primary atom site location: structure-invariant direct methods	Extinction correction: none

### Special details

**Table:**

Geometry. All e.s.d.'s (except the e.s.d. in the dihedral angle between two l.s. planes) are estimated using the full covariance matrix. The cell e.s.d.'s are taken into account individually in the estimation of e.s.d.'s in distances, angles and torsion angles; correlations between e.s.d.'s in cell parameters are only used when they are defined by crystal symmetry. An approximate (isotropic) treatment of cell e.s.d.'s is used for estimating e.s.d.'s involving l.s. planes.
Refinement. Refinement of *F*^2^ against ALL reflections. The weighted *R*-factor *wR* and goodness of fit *S* are based on *F*^2^, conventional *R*-factors *R* are based on *F*, with *F* set to zero for negative *F*^2^. The threshold expression of *F*^2^ > σ(*F*^2^) is used only for calculating *R*-factors(gt) *etc*. and is not relevant to the choice of reflections for refinement. *R*-factors based on *F*^2^ are statistically about twice as large as those based on *F*, and *R*- factors based on ALL data will be even larger.

### Fractional atomic coordinates and isotropic or equivalent isotropic displacement parameters (Å^2^)

**Table:**

	*x*	*y*	*z*	*U*_iso_*/*U*_eq_	
C1	−0.1339 (2)	0.7322 (2)	1.1693 (2)	0.0306 (5)	
C2	−0.3294 (2)	0.6903 (2)	1.0760 (2)	0.0329 (5)	
H2	−0.3880	0.6499	0.9555	0.039*	
C3	−0.4385 (2)	0.7073 (2)	1.1574 (3)	0.0359 (5)	
H3	−0.5721	0.6780	1.0931	0.043*	
C4	−0.3540 (3)	0.7668 (2)	1.3323 (3)	0.0412 (5)	
H4	−0.4296	0.7781	1.3881	0.049*	
C5	−0.1609 (3)	0.8098 (2)	1.4261 (3)	0.0428 (5)	
H5	−0.1030	0.8523	1.5465	0.051*	
C6	−0.0511 (3)	0.7910 (2)	1.3450 (2)	0.0395 (5)	
H6	0.0823	0.8186	1.4101	0.047*	
C7	−0.0110 (3)	0.7152 (2)	1.0855 (3)	0.0382 (5)	
O7	0.15653 (19)	0.7341 (2)	1.16374 (19)	0.0670 (5)	
C8	−0.1064 (2)	0.6727 (3)	0.8971 (2)	0.0435 (6)	
H8B	−0.2071	0.5732	0.8425	0.052*	
H8A	−0.1661	0.7559	0.8701	0.052*	
O9	0.02347 (17)	0.65426 (17)	0.82904 (17)	0.0437 (4)	
C10	0.1269 (3)	0.7883 (3)	0.8448 (3)	0.0385 (5)	
O10	0.1180 (2)	0.91629 (19)	0.9139 (2)	0.0649 (5)	
C11	0.2487 (3)	0.7577 (3)	0.7621 (2)	0.0313 (5)	
C12	0.2426 (2)	0.6074 (2)	0.6700 (2)	0.0320 (5)	
H12	0.1609	0.5172	0.6585	0.038*	
C13	0.3593 (2)	0.5930 (2)	0.5953 (2)	0.0364 (5)	
H13	0.3537	0.4900	0.5309	0.044*	
N14	0.4789 (2)	0.7134 (2)	0.6074 (2)	0.0396 (5)	
C15	0.4825 (3)	0.8565 (3)	0.6977 (3)	0.0420 (5)	
H15	0.5670	0.9447	0.7090	0.050*	
C16	0.3708 (3)	0.8837 (2)	0.7753 (2)	0.0383 (5)	
H16	0.3779	0.9881	0.8372	0.046*	

### Atomic displacement parameters (Å^2^)

**Table:**

	*U*^11^	*U*^22^	*U*^33^	*U*^12^	*U*^13^	*U*^23^
C1	0.0319 (10)	0.0331 (13)	0.0341 (12)	0.0088 (9)	0.0194 (9)	0.0165 (11)
C2	0.0308 (10)	0.0375 (13)	0.0332 (12)	0.0093 (10)	0.0170 (10)	0.0140 (11)
C3	0.0365 (11)	0.0362 (13)	0.0503 (14)	0.0156 (10)	0.0285 (11)	0.0220 (12)
C4	0.0579 (13)	0.0397 (14)	0.0509 (15)	0.0211 (12)	0.0400 (12)	0.0257 (13)
C5	0.0564 (14)	0.0438 (14)	0.0335 (12)	0.0140 (12)	0.0244 (12)	0.0165 (12)
C6	0.0389 (12)	0.0429 (14)	0.0373 (13)	0.0097 (11)	0.0170 (11)	0.0176 (12)
C7	0.0326 (11)	0.0475 (14)	0.0450 (13)	0.0155 (11)	0.0221 (11)	0.0238 (12)
O7	0.0326 (8)	0.1257 (16)	0.0589 (10)	0.0276 (10)	0.0249 (8)	0.0486 (12)
C8	0.0364 (11)	0.0638 (16)	0.0409 (13)	0.0164 (12)	0.0272 (11)	0.0188 (13)
O9	0.0439 (8)	0.0525 (10)	0.0496 (9)	0.0144 (8)	0.0353 (8)	0.0186 (9)
C10	0.0416 (12)	0.0492 (15)	0.0369 (13)	0.0197 (12)	0.0232 (11)	0.0225 (13)
O10	0.0978 (12)	0.0517 (11)	0.0899 (13)	0.0419 (10)	0.0749 (11)	0.0320 (11)
C11	0.0336 (10)	0.0400 (12)	0.0291 (11)	0.0155 (10)	0.0188 (9)	0.0163 (11)
C12	0.0328 (10)	0.0362 (13)	0.0321 (11)	0.0091 (10)	0.0192 (10)	0.0133 (11)
C13	0.0422 (11)	0.0408 (14)	0.0342 (12)	0.0177 (11)	0.0229 (10)	0.0144 (11)
N14	0.0452 (10)	0.0467 (13)	0.0401 (11)	0.0170 (10)	0.0269 (9)	0.0216 (10)
C15	0.0492 (12)	0.0411 (14)	0.0425 (13)	0.0081 (12)	0.0264 (12)	0.0188 (12)
C16	0.0501 (12)	0.0354 (13)	0.0390 (12)	0.0159 (11)	0.0274 (11)	0.0153 (11)

### Geometric parameters (Å, °)

**Table:**

C1—C6	1.387 (2)	C8—H8B	0.990
C1—C2	1.390 (2)	C8—H8A	0.990
C1—C7	1.495 (2)	O9—C10	1.343 (2)
C2—C3	1.378 (2)	C10—O10	1.196 (2)
C2—H2	0.950	C10—C11	1.488 (2)
C3—C4	1.380 (3)	C11—C16	1.376 (2)
C3—H3	0.950	C11—C12	1.386 (3)
C4—C5	1.374 (3)	C12—C13	1.384 (2)
C4—H4	0.950	C12—H12	0.950
C5—C6	1.381 (2)	C13—N14	1.330 (2)
C5—H5	0.950	C13—H13	0.950
C6—H6	0.950	N14—C15	1.334 (2)
C7—O7	1.204 (2)	C15—C16	1.375 (2)
C7—C8	1.499 (3)	C15—H15	0.950
C8—O9	1.4371 (19)	C16—H16	0.950
			
C6—C1—C2	118.97 (16)	O9—C8—H8A	109.2
C6—C1—C7	119.22 (17)	C7—C8—H8A	109.2
C2—C1—C7	121.80 (17)	H8B—C8—H8A	107.9
C3—C2—C1	120.25 (18)	C10—O9—C8	115.55 (15)
C3—C2—H2	119.9	O10—C10—O9	123.68 (17)
C1—C2—H2	119.9	O10—C10—C11	124.4 (2)
C2—C3—C4	120.10 (18)	O9—C10—C11	111.85 (18)
C2—C3—H3	119.9	C16—C11—C12	118.45 (16)
C4—C3—H3	119.9	C16—C11—C10	118.77 (19)
C5—C4—C3	120.23 (17)	C12—C11—C10	122.77 (19)
C5—C4—H4	119.9	C13—C12—C11	117.82 (17)
C3—C4—H4	119.9	C13—C12—H12	121.1
C4—C5—C6	119.85 (19)	C11—C12—H12	121.1
C4—C5—H5	120.1	N14—C13—C12	124.53 (18)
C6—C5—H5	120.1	N14—C13—H13	117.7
C5—C6—C1	120.57 (18)	C12—C13—H13	117.7
C5—C6—H6	119.7	C13—N14—C15	116.29 (15)
C1—C6—H6	119.7	N14—C15—C16	123.75 (19)
O7—C7—C1	122.35 (18)	N14—C15—H15	118.1
O7—C7—C8	120.97 (16)	C16—C15—H15	118.1
C1—C7—C8	116.67 (15)	C15—C16—C11	119.15 (18)
O9—C8—C7	112.06 (15)	C15—C16—H16	120.4
O9—C8—H8B	109.2	C11—C16—H16	120.4
C7—C8—H8B	109.2		
			
C6—C1—C2—C3	0.0 (3)	C8—O9—C10—O10	1.7 (3)
C7—C1—C2—C3	179.89 (17)	C8—O9—C10—C11	−176.62 (16)
C1—C2—C3—C4	0.3 (3)	O10—C10—C11—C16	5.1 (3)
C2—C3—C4—C5	0.2 (3)	O9—C10—C11—C16	−176.53 (18)
C3—C4—C5—C6	−1.0 (3)	O10—C10—C11—C12	−173.9 (2)
C4—C5—C6—C1	1.4 (3)	O9—C10—C11—C12	4.4 (3)
C2—C1—C6—C5	−0.9 (3)	C16—C11—C12—C13	−0.5 (3)
C7—C1—C6—C5	179.23 (19)	C10—C11—C12—C13	178.58 (17)
C6—C1—C7—O7	8.4 (3)	C11—C12—C13—N14	0.9 (3)
C2—C1—C7—O7	−171.5 (2)	C12—C13—N14—C15	−0.5 (3)
C6—C1—C7—C8	−171.7 (2)	C13—N14—C15—C16	−0.3 (3)
C2—C1—C7—C8	8.4 (3)	N14—C15—C16—C11	0.6 (3)
O7—C7—C8—O9	1.8 (3)	C12—C11—C16—C15	−0.2 (3)
C1—C7—C8—O9	−178.06 (16)	C10—C11—C16—C15	−179.31 (19)
C7—C8—O9—C10	−80.8 (2)		

### Hydrogen-bond geometry (Å, °)

**Table:**

*D*—H···*A*	*D*—H	H···*A*	*D*···*A*	*D*—H···*A*
C4—H4···CgB^i^	0.95	3.86	4.766 (3)	160
C2—H2···CgB^ii^	0.95	2.89	3.640 (3)	137
C6—H6···CgB^iii^	0.95	3.00	3.752 (3)	137
C12—H12···CgA^iv^	0.95	2.88	3.572 (3)	130

Symmetry codes: (i) *x*−1, *y*, *z*+1; (ii) *x*−1, *y*, *z*; (iii) *x*, *y*, *z*+1; (iv) −*x*, −*y*+1, −*z*+2.
